# A role for AKT1 in nonsense-mediated mRNA decay

**DOI:** 10.1093/nar/gkab882

**Published:** 2021-10-11

**Authors:** Martine Palma, Catherine Leroy, Sophie Salomé-Desnoulez, Elisabeth Werkmeister, Rebekah Kong, Marc Mongy, Hervé Le Hir, Fabrice Lejeune

**Affiliations:** Univ. Lille, CNRS, Inserm, CHU Lille, UMR9020-U1277 - CANTHER - Cancer Heterogeneity Plasticity and Resistance to Therapies, F-59000 Lille, France; Unité tumorigenèse et résistance aux traitements, Institut Pasteur de Lille, F-59000 Lille, France; Univ. Lille, CNRS, Inserm, CHU Lille, UMR9020-U1277 - CANTHER - Cancer Heterogeneity Plasticity and Resistance to Therapies, F-59000 Lille, France; Unité tumorigenèse et résistance aux traitements, Institut Pasteur de Lille, F-59000 Lille, France; Univ. Lille, CNRS, Inserm, CHU Lille, Institut Pasteur de Lille, US 41 - UMS 2014 - PLBS, F-59000 Lille, France; Univ. Lille, CNRS, Inserm, CHU Lille, Institut Pasteur de Lille, US 41 - UMS 2014 - PLBS, F-59000 Lille, France; Univ. Lille, CNRS, Inserm, CHU Lille, Institut Pasteur de Lille, U1019 – UMR9017 – CIIL – center for Infection and Immunity of Lille, F-59000 Lille, France; Univ. Lille, CNRS, Inserm, CHU Lille, UMR9020-U1277 - CANTHER - Cancer Heterogeneity Plasticity and Resistance to Therapies, F-59000 Lille, France; Unité tumorigenèse et résistance aux traitements, Institut Pasteur de Lille, F-59000 Lille, France; Univ. Lille, CNRS, Inserm, CHU Lille, Institut Pasteur de Lille, US 41 - UMS 2014 - PLBS, F-59000 Lille, France; Institut de Biologie de l’Ecole Normale Supérieure (IBENS), Ecole Normale Supérieure, CNRS, INSERM, PSL Research University, 46 rue d’Ulm, 75005 Paris, France; Univ. Lille, CNRS, Inserm, CHU Lille, UMR9020-U1277 - CANTHER - Cancer Heterogeneity Plasticity and Resistance to Therapies, F-59000 Lille, France; Unité tumorigenèse et résistance aux traitements, Institut Pasteur de Lille, F-59000 Lille, France

## Abstract

Nonsense-mediated mRNA decay (NMD) is a highly regulated quality control mechanism through which mRNAs harboring a premature termination codon are degraded. It is also a regulatory pathway for some genes. This mechanism is subject to various levels of regulation, including phosphorylation. To date only one kinase, SMG1, has been described to participate in NMD, by targeting the central NMD factor UPF1. Here, screening of a kinase inhibitor library revealed as putative NMD inhibitors several molecules targeting the protein kinase AKT1. We present evidence demonstrating that AKT1, a central player in the PI3K/AKT/mTOR signaling pathway, plays an essential role in NMD, being recruited by the UPF3X protein to phosphorylate UPF1. As AKT1 is often overactivated in cancer cells and as this should result in increased NMD efficiency, the possibility that this increase might affect cancer processes and be targeted in cancer therapy is discussed.

## INTRODUCTION

Quality controls occur at different steps in gene expression. One such control is nonsense-mediated mRNA decay (NMD), which prevents the synthesis of potentially deleterious truncated proteins by targeting mRNAs carrying a premature termination codon (PTC) (1–4). NMD involves more than a dozen factors, including the central factors UPF1, UPF2 and UPF3X (also named UPF3B). UPF1 and UPF2 are phosphoproteins (5,6). Phosphorylation of both proteins is required for NMD activation, and this suggests that kinases involved in their phosphorylation may regulate NMD. Although the role of UPF2 phosphorylation needs to be investigated, UPF1 phosphorylation has been abundantly studied (7–12). For instance, UPF1 phosphorylation at the Threonine 28 promotes the recruitment of SMG6, while phosphorylation of the Serine 1096 induces the recruitment of SMG5/SMG7 to NMD mRNP targets, causing departure of the ribosome paused on the PTC and activation of mRNA decay pathways (6,13,14). In addition, phospho-UPF1 impairs the function of eIF3, required to initiate new rounds of translation on the PTC-containing mRNAs (15). To date, the only kinase shown to be involved in NMD by directly targeting an NMD factor is SMG1, a phosphatidylinositol 3-kinase-related protein kinase shown to phosphorylate UPF1 (16–18). As UPF2 is also a phosphoprotein (5,19) and as no UPF2-phosphorylating kinase has been identified in vivo, it is strongly expected that additional kinases are involved in NMD.

AKT (also called protein kinase B) is a serine/threonine kinase involved in various cellular processes such as cell cycle progression, glucose metabolism, cell proliferation, translation, and transcription. Accordingly, this protein localizes to the cytoplasm and nucleus (20,21). The role of AKT in cancer is very complex. The *AKT* gene is viewed as an oncogene, as it is often overexpressed in cancer. AKT, furthermore, promotes cell proliferation and also plays an anti-apoptotic role by inhibiting pro-apoptotic proteins (22). AKT exists in three different isoforms, named AKT1, AKT2 and AKT3. These isoforms are differentially expressed according to the tissue and developmental stage (23,24). Whether they are functionally redundant and can replace each other is unclear.

Here, we present evidence that AKT1 is involved in NMD by specifically phosphorylating UPF1 but not UPF2. We further show that AKT1 is recruited to mRNPs by UPF3X before interacting with UPF1. The involvement of AKT1 in NMD appears as essential as that of the SMG1 protein, since the absence of either protein causes inhibition of NMD and since no cumulative effect is observed in the absence of both proteins. Given the role of AKT1 in tumorigenesis, this discovery may foreshadow a role of NMD in the cancer process.

## MATERIALS AND METHODS

### Screening assay

The screening method used has been described previously (25). The kinase inhibitor library is described in (26). Molecules were tested at 10 μM in DMSO.

### Cell culture

HeLa cells were grown in DMEM supplemented with 10% FBS and 1% Zell Shield (Minerva Biolabs, Berlin, Germany) at 37°C under 5% CO_2_. HEK293FT WT and HEK293FT ΔAKT1 cells were grown at 37°C and 5% CO_2_ in DMEM supplemented with 10% non-heat inactivated FBS, 1% Zell Shield.

To obtain the HEK293FT ΔAKT1 cells with the CRISPR/Cas9 system, we used the pLentiV2-CRISPR plasmid (a gift from Feng Zhang, Addgene plasmid # 52961; http://n2t.net/addgene:52961; RRID:Addgene_52961) (27) with gRNA complementing a sequence in the *AKT1* gene (5′-CACCGCCCGCGCACGCTTGGTCCCG-3′). Cells were selected with 3 μg/ml puromycin (InvivoGen).

### Cell proliferation Assay

HEK293FT WT and HEK293FT ΔAKT1 cells (3 × 10^3^ per well) were seeded in duplicate into 96-well plates and grown in 150 μl supplemented DMEM. Every two days, the medium was changed.

The IncuCyte^®^ live-cell imaging and analysis system was used and cell proliferation was monitored by analyzing the occupied area (% confluence) on cell images over 130 hours of culture.

The images obtained were taken with a 4x objective lens every 2 h.

### Chemicals

The AKT1 inhibitor was purchased from CliniSciences (ApexBio A-674563). The molecule was dissolved in DMSO and used at 800 nM.

### Plasmids, siRNA, and transfection

Plasmid pcDNA3 flag HA AKT1 was a gift from William Sellers (Addgene plasmid # 9021; http://n2t.net/addgene:9021; RRID: Addgene_9021) (28). The pcDNA3-HA-AKT1-K179M expression vector expressing an inactive mutant form of AKT1 (altered kinase domain) was a gift from Jie Chen (Addgene plasmid #73409; http://n2t.net/addgene:73409; RRID: Addgene_73409) (29). Plasmid mCherry-AKT1 E17K, causing constitutive activation of AKT1, was a generous gift from Dr Anne-Laure Todeschini. Cells were transfected with Jet Optimus Reagent (Polyplus Transfection Ref: 117-01).

With the AKT1 inhibitor, Lipofectamine 3000 (Life Technologies) was used according to the supplier's recommendations. ICAFectin™ 442 reagent (In Cell Art, Nantes, France) was used to transfect HEK293FT WT and ΔAKT1 cells with a control siRNA (Eurogentec), siRNA UPF1 (5′-AAGATGCAGTTCCGCTCCATTTT-3′), siRNA UPF2 (5′-GAAGTTGGTACGGGCACTC-3′), siRNA UPF3X (5′-GGAGAAGCGAGTAACCCTG-3′) (Sigma Aldrich), siRNA AKT1 (5′-GAAGGAAGUCAUCGUGGCCAA-3′), siRNA AKT2 (5′- CUCUUCGAGCUCAUCCUCA-3′), siRNA AKT3 (5′-GAAAGAUUGUGUACCGUGA-3′), siRNA SMG1 (5′- CCAGGACACGAGGAAACUG-3′) and pmCMV-Gl Norm or pmCMV-Gl Ter and pIE-MUP].

### Protein extraction and western blotting

Proteins were extracted in the following lysis buffer: 50 mM Tris–HCl pH 7.4, 20 mM EDTA pH 8, 5% SDS from about 2 million of cells. After 30 pulses of sonication (Branson Digital Sonifier/amplitude 20%), proteins were analyzed by western blotting. Migration of all proteins was carried out in a 6%, 10% or 12% SDS-PAGE gel. After migration, the proteins were transferred to a nitrocellulose membrane and incubated with primary antibodies overnight at 4°C before incubation with a secondary antibody (Jackson ImmunoResearch, Baltimore-Pike, PA, USA) 111-035-003 (rabbit) or 115-035-003 (mouse)) for 1 h at room temperature. The proteins were observed with SuperSignal West Femto Maximum Sensitivity Substrate (Pierce-Biotechnology, Rockford, IL, USA). The primary antibodies used were: rabbit anti- AKT1 antibody at 1:1000 (Cell Signaling #2938), rabbit anti-Akt2 antibody (5B5) at 1:500 (Cell Signaling #2964), rabbit anti-Akt3 antibody (62A8) at 1:500 (Cell Signaling #3788), rabbit anti-UPF1 antibody at 1:5000 (Abcam ab86057), rabbit anti-UPF2 antibody at 1:1000 (Eurogentec), rabbit anti-UPF3X antibody at 1:2000 (Abcam ab134566), rabbit anti-importin 9 at 1:1000 (Abcam ab52605), mouse anti- eIF4E antibody at 1:500 (Santa Cruz Biotechnology sc-9976), rabbit anti-Phospho AKT1 antibody at 1:1000 (Abcam ab133458), rabbit anti-Phospho AKT antibody at 1:2000 (Cell Signaling #4060), rabbit anti-MDM2 antibody at 1:1000 (Abcam ab16895), rabbit anti-CBP80 antibody (H-300) at 1:1000 (Santa Cruz Biotechnology sc-48803), rabbit anti-phospho Ser/Thr ATM/ATR Substrate antibody at 1:1000 (Cell Signaling #2851), rabbit anti-phospho UPF1/Rent1 Thr28 antibody (Biorbyt orb7836), rabbit anti-SMG1 antibody at 1:500 (Abcam ab30916).

### RNA extraction and RT-PCR

Total RNA was extracted from about 2 million of cell using RNazol reagent and according to the manufacturer protocol. RT-PCR was performed as described (25). The primer sequences used in this study were for: MUP (sense 5′-CTGATGGGGCTCTATG-3′; antisense 5′-TCCTGGTGAGAAGTCTCC-3′) or Globin (sense 5′-GGACGAGCTGTACAAGTATC-3′; antisense 5′-GGGTTTAGTGGTACTTGTGAGC-3′).

### *In vitro* phosphorylation assay

The *in vitro* phosphorylation assay was carried out with the ADP-Glo™ Kinase Assay kit (Promega; V6930) and the AKT1 Kinase Enzyme System Kit (V1911). The purified proteins UPF1, and UPF2 (761-1227) which includes the three phosphorylation sites (S886, S992 and S1046) identified by mass spectrometry (19) were obtained from Dr Hervé Le Hir. The UPF3X protein was purchased from CliniSciences (*Recombinant Human Regulator of Nonsense Transcripts 3B—E. coli*; CSB-EP883646HU). Kinase reactions were carried out at room temperature for 1 h with 50 ng enzyme, 1 μg substrate, purified protein, 250 μM ATP, 50 μM DTT, Reaction Buffer 1×. The ATP depletion reaction was carried out at room temperature for 40 min and the luminescence reaction was carried out for 30 min in a Tristar luminometer (Berthold).

### Immunoprecipitations

For immunoprecipitation of UPF1 without stimulation of NMD and for immunoprecipitation of UPF3X, 160 million of HEK293FT cells were lysed in lysis buffer containing: 50 mM Tris-HCl pH 7.4, 300 mM NaCl, 0.05% NP40, and Halt™ protease, phosphatase inhibitor cocktail (Thermo Scientific). After 30 pulses of sonication (Branson Digital Sonifier/amplitude 20%), the cell extracts were incubated with rabbit anti-UPF1 antibody (Abcam ab86057) or rabbit anti-UPF3X antibody (Abcam ab134566). After 2 h at 4°C, protein A agarose beads were added to the cell extracts and incubated for 1 h at 4°C. The beads were then washed five times with lysis buffer before eluting proteins from beads with 2× sample loading buffer (0.1 M Tris–HCl (pH 6.8), 4% SDS, 12% β-mercaptoethanol, 20% glycerol, bromophenol blue).

For immunoprecipitation of UPF1 with stimulation of NMD, calcium chloride was used to transfect HEK293FT cells with Globin Ter plasmid. After transfection, the preparation of cell extracts was similar to the protocol described above.

For immunoprecipitation of AKT1, another lysis buffer was used: 25 mM Tris pH 7.5, 150 mM NaCl, 1 mM EDTA, 1% Triton X100. The washing buffer used was composed of 20 mM Tris pH 7.5, 50 mM NaCl, 5 mM EDTA and 0.1% Triton x100. The same protocol steps were followed.

For immunoprecipitation of AKT1 with downregulation of *UPF1* or *UPF3X* expression, ICAFectin™ 442 reagent was used to transfect HEK293FT cells with siRNA. The nuclear and cytoplasmic fractions were obtained with the NE-PER™ Nuclear and Cytoplasmic extraction kit (Thermo Scientific 78835) according to the manufacturer's recommendations. The lysis and washing buffers used were the same as for immunoprecipitation of AKT1.

For immunoprecipitations in the presence or absence of RNase A, cell extracts were incubated for 30 min at 37°C. with 10 μg BSA or RNase A before incubation with the antibodies.

### Proximity ligation assay

The proximity ligation assay was performed with the kit from Sigma—*Duolink™ In Situ Orange Starter Kit Mouse/Rabbit* (Ref: DUO92102) with λ_ex_ 554 nm; λ_em_ 576 nm (Cyanine 3; Zeiss Filter set 20) according to the manufacturer's recommendations. The Nunc™ Lab-Tek II Chamber Slide (Thermo Scientific Nunc, Ref: 154534) was used for the proximity ligation assay. The primary antibodies used were: rabbit anti-UPF1 antibody at 1:250 (Abcam ab86057), rabbit anti-UPF2 antibody at 1:250 (Eurogentec), rabbit anti-UPF3X antibody at 1:250 (Abcam ab134566), mouse anti-AKT1 antibody at 1:50 (Santa Cruz sc-271149), mouse anti-eIF4E antibody at 1:250 (Santa Cruz sc-9976), rabbit anti-MDM2 antibody at 1:250 (Cell Signaling #86934).

### Image analysis and quantification

Live imaging was performed with the Spinning Disk- Live SR microscope (Ti2 Nikon—Spinning Disk Yokogawa CSUW1—Gataca) with Metamorph software. Images were taken at different wavelengths (excitation: 405, 561, and 488 nm; emission filters 450/50, 525/50 and 595/50). The SR module was used to improve the resolution. Observations were done with a 60× oil immersion objective (Nikon Plan Apo 60× NA 1.4). Images were processed with the Huygens Professional Software. After the deconvolution, the images were first analyzed with the ImageJ software (NIH) and then with Imaris (Bitplane version 9.5.0) in order to count the number of spots in each image (spot detection module).

## RESULTS

### Screening of a kinase inhibitor library reveals AKT1 as a putative NMD factor

**Figure 1.**
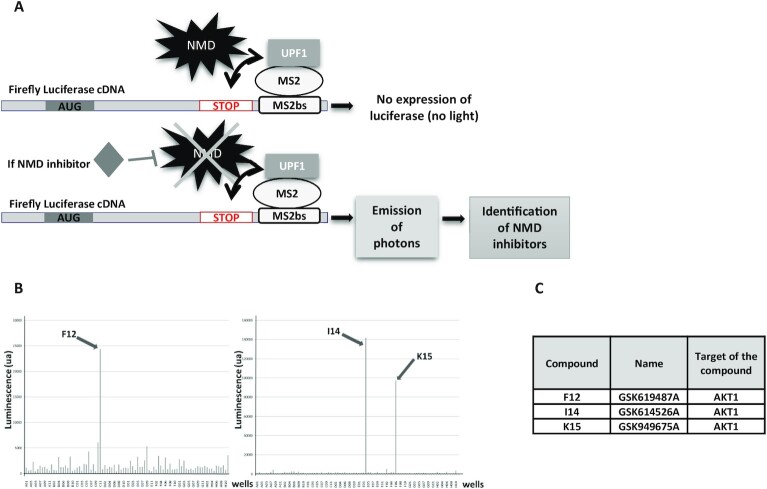
Screening of the PKIS library. (**A**) Schematic representation of the screening system used to identify NMD inhibitors. HeLa cells are transfected with constructs expressing the cDNA encoding the firefly luciferase carrying MS2 binding sites in the 3′UTR and expressing an MS2/UPF1 fusion protein. If a tested molecule inhibits NMD, the firefly luciferase mRNA is stabilized and translated to a functional firefly luciferase, the activity of which is measurable. (**B**) Results of the screening of the two plates containing the molecules promoting the highest luciferase activity. (**C**) Molecules and target identification.

To identify new kinases involved in NMD, a kinase inhibitor library was screened. For this we used a construct carrying the open reading frame encoding the firefly luciferase followed, in the 3′UTR, by MS2 binding sequences (Figure 1A) (25). This construct was co-expressed with a cDNA encoding an MS2/UPF1 fusion protein. The goal was to identify kinases targeting the known phosphoprotein UPF1 (8,30). The screened library, named PKIS, consisted of 367 kinase inhibitors from the company GlaxoSmithKline (26). The screen revealed three kinase inhibitors promoting a very high level of luciferase activity, suggesting that they could strongly inhibit NMD (Figure 1B). Interestingly, all three selected compounds (GSK619487A, GSK614526A and GSK949675A) were designed to target the same kinase: the AKT1 protein (Figure 1C). This result strongly suggests that AKT1 plays a role in NMD. It should be noted that this library does not contain molecules designed to inhibit the SMG1 protein.

### NMD is inhibited in cells lacking AKT1 activity

**Figure 2.**
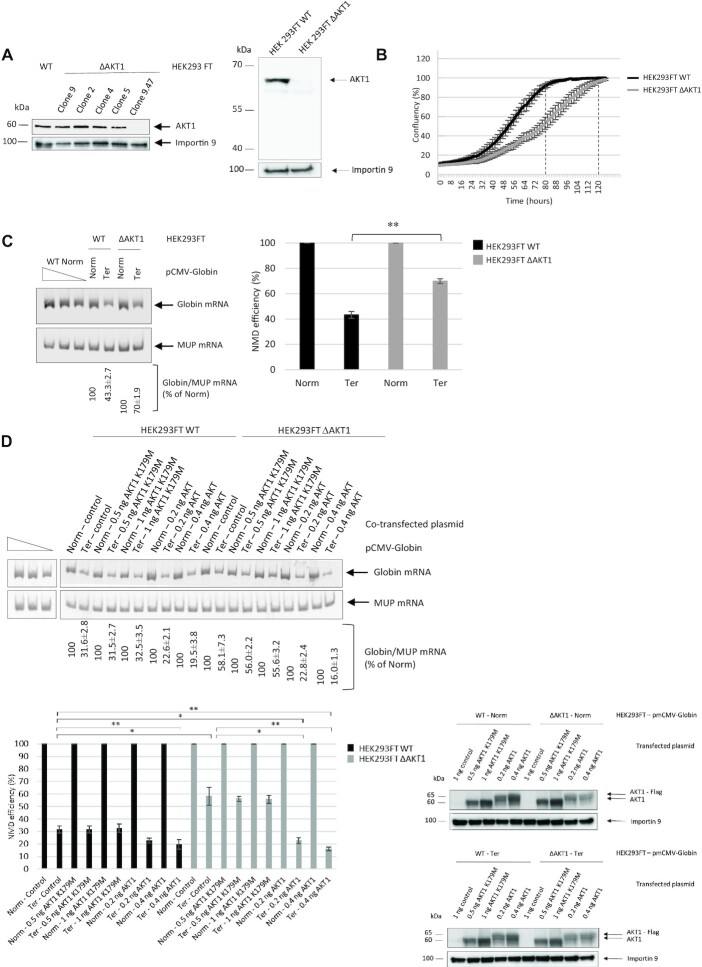
The absence of AKT1 impairs NMD. (**A** left panel) Western blot showing levels of AKT1 protein in various HEK293FT ΔAKT1 clones. Importin 9 was used as a loading control. (right panel) Western-blot analysis of AKT1 from HEK293FT WT and HEK293FT ΔAKT1 cells, showing the absence of AKT1 or truncated AKT1 expression in HEK293FT ΔAKT1 cells. (**B**) Growth curves of HEK293FT WT cells (in black) and HEK293FT ΔAKT1 cells (in grey). Percent confluence was measured as a function of time. The two broken lines indicate the confluence points of the two cell lines. (**C**) Measure of NMD efficiency in the presence and absence of AKT1. The left panel represents levels of WT and PTC-carrying globin mRNAs as measured by RT-PCR. MUP mRNA was used as a loading control. The three leftmost lanes correspond to serial dilutions of a Globin Norm sample from WT cells. The bar plot on the right shows the measured NMD efficiency. (**D**) The kinase activity of AKT1 is required for its role in NMD. The NMD efficiency was measured as in (**C**) in cells overexpressing AKT1 or AKT1-K179M. The lower right panels show a western blot analysis of AKT1 and AKT1-K179M levels in cells transfected with the corresponding expression vector. The level of AKT1 was evaluated in HEK293FT WT and HEK293FT ΔAKT1 cells after transfection with pmCMV-Globin Norm, pmCMV-Globin Ter, pmCMV (control), pAKT1-K179M or pAKT1. Importin 9 was used as a loading control. The results of Figure 2 are representative of at least two experiments. Error bar = S.D., *P*-values were calculated with Student's *t*-test: *<0.05, **<0.01.

To validate the screening results, CRISPR-Cas9 technology was used in HEK293FT cells to impair *AKT1* expression. Several clones were isolated and clone 9.47 was selected for further use on the basis of a western blot analysis demonstrating its low residual level of AKT1 protein (Figure 2A). In what follows, cells of this clone are called HEK293FT ΔAKT1 cells. To exclude the possibility of causing synthesis of a truncated AKT1 protein through use of CRISPR/Cas9 technology, we carried out a western blot analysis allowing the detection of a small protein migrating faster than AKT1. This analysis did not show any evidence of the presence of a truncated protein (Figure 2A right panel). The HEK293FT ΔAKT1 cells were found to proliferate more slowly than wild-type HEK293FT cells because of the loss of AKT1 function (Figure 2B). The time required to reach the confluence plateau was about 120 hours for HEK293FT ΔAKT1 cells versus 80 hours for wild-type cells.

To assess whether AKT1 is required for NMD, wild-type and HEK293FT ΔAKT1 cells were transfected with a plasmid expressing wild-type globin mRNA (called Globin Norm), a PTC-carrying globin mRNA (called Globin Ter), or MUP mRNA as a control (Figure 2C). The absence of AKT1 appeared to promote inhibition of NMD, since the level of Globin Ter mRNA was significantly (about 1.6-fold) higher in HEK293FT ΔAKT1 cells than in wild-type HEK293FT cells. Interestingly, this NMD inhibition was also observed when A-674563, a chemical inhibitor of AKT1 (31), was added at 800 nM for 10 h to the culture medium of wild-type or HEK293FT ΔAKT1 cells (Supplementary Figure S1). Strong inhibition of AKT1 phosphorylation by this treatment was demonstrated by western blotting blotting (Supplementary Figure S1A).

To rule out the possibility that the inhibition of NMD shown in Figure 2C might have been a nonspecific consequence of impairing the *AKT1* gene with CRISPR/Cas9, the wild-type AKT1 protein was reintroduced into HEK293FT ΔAKT1 cells by transient transfection with an expression vector encoding WT AKT1 with a Flag tag at the N-terminal end. The level of AKT1 protein was monitored by western blotting and the amount of transfecting vector was adjusted, on the basis of western blot data, so as to have similar levels of AKT1 under the different test conditions (Figure 2D). The level of NMD inhibition was then measured by RT-PCR (Figure 2D). In both wild-type and HEK293FT ΔAKT1 cells, the additional amount of AKT1 generated from the transfecting plasmid resulted in a significant increase in NMD efficiency. In HEK293FT ΔAKT1 cells, the AKT1 synthesized from the plasmid restored the NMD efficiency found in wild-type cells. This result confirms that the loss of AKT1 is responsible for the inhibition of NMD observed in HEK293FT ΔAKT1 cells. To exclude the possibility that the inhibition of NMD observed in the absence of AKT1 might be due to a general inhibition of translation, we measured the activity of the firefly luciferase from the same number of HEK293FT WT and HEK293FT ΔAKT1 cells transfected with a construct encoding firefly luciferase that resulted in the absence of a significant decrease of the translation rate in the absence of AKT1 under our experimental conditions (Supplementary Figure S2). To determine whether NMD inhibition might be due to loss of the AKT1 kinase activity, cells were transfected with a plasmid encoding a Flag-tagged mutated version of AKT1 in which the kinase domain is inactivated because of the K179M mutation (29). With this mutated version of AKT1, the NMD efficiency was not restored to the wild-type level and remained similar to that observed under control conditions. This indicates that the kinase activity of AKT1 is necessary to rescue NMD efficiency in HEK293FT ΔAKT1 cells (Figure 2D).

Overall, these results demonstrate that AKT1, via its kinase activity, is involved in NMD. Since there are three AKT isoforms whose functions might partially overlap, the effects of the other two AKT isoforms were tested in the NMD reaction. For this, *AKT2* or *AKT3* expression was impaired with siRNA and the levels of Globin Norm and Globin Ter mRNAs were measured in transfected cells (Supplementary Figure S3). Downregulation of *AKT2* or *AKT3* expression did not promote inhibition of NMD. This indicates that AKT1 alone is involved in NMD.

### AKT1 interacts with UPF proteins

**Figure 3.**
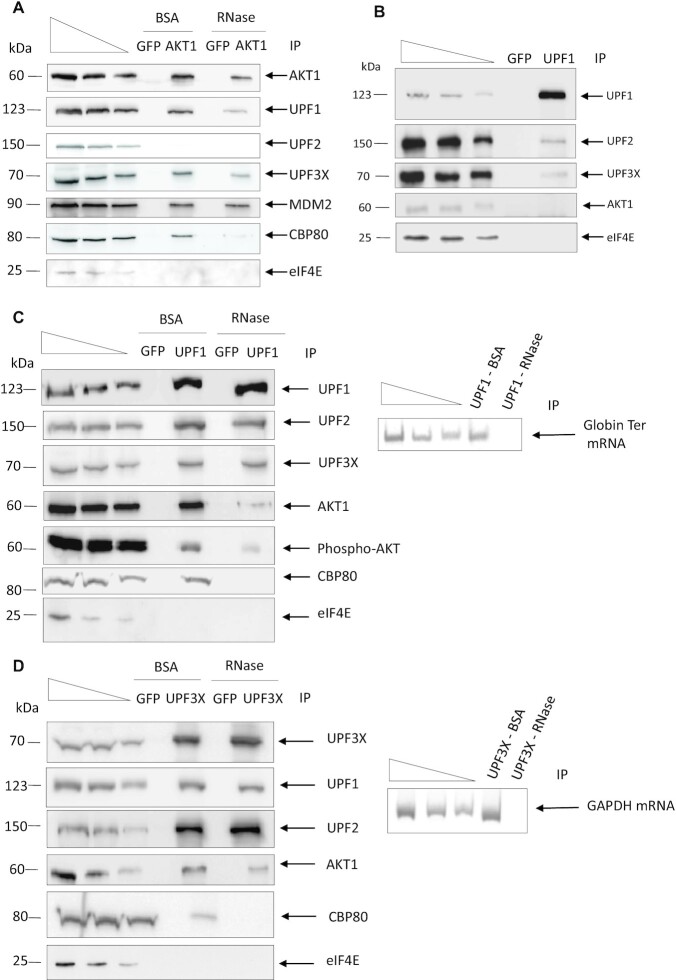
AKT1 interacts with NMD factors. (**A**) AKT1 immunoprecipitation analysis. AKT1 was immunoprecipitated from HEK293FT WT cells. The immunoprecipitations were performed in the presence of RNase A or BSA to assess the requirement for RNA in the protein interactions. The immunoprecipitate was then analyzed by western blotting for the presence of co-immunoprecipitated factors. MDM2 as a partner of AKT1 was used as positive control. (**B**) UPF1 immunoprecipitation analysis. UPF1 was immunoprecipitated from HEK293FT WT cells and the immunoprecipitate analyzed by western blotting for the presence of co-immunoprecipitated factors. (**C**) UPF1 immunoprecipitation analysis under NMD activation. UPF1 was immunoprecipitated from HEK293FT WT cells transfected with a construct expressing PTC-carrying globin mRNA in the presence of RNase A or BSA to assess the requirement for RNA in the protein interactions. The immunoprecipitate was then analyzed by western blotting for the presence of co-immunoprecipitated factors. The right panel is the result of an RT-PCR performed on RNA extracted from the immunoprecipitates to assess the efficiency of the RNase A treatment. (**D**) UPF3X immunoprecipitation analysis. UPF3X was immunoprecipitated from HEK293FT WT cells and the immunoprecipitate analyzed by western blotting for the presence of co-immunoprecipitated factors. GFP immunoprecipitation was performed as a negative control to assess the specificity of the immunoprecipitations. The immunoprecipitations were performed in the presence of RNase A or BSA to assess the requirement for RNA in the protein interactions. The immunoprecipitate was then analyzed by western blotting for the presence of co-immunoprecipitated factors. The right panel is the result of RT-PCR performed on RNA extracted from the immunoprecipitates to assess the efficiency of RNase A treatment. The three leftmost lanes in each panel of the figure correspond to serial dilutions of HEK293FT whole cell extract. The results presented in this figure are representative of two experiments.

The involvement of the kinase activity of AKT1 in NMD suggests that AKT1 may phosphorylate certain NMD factors and thus interact with proteins of the NMD mechanism. To test this hypothesis, endogenous AKT1 was immunoprecipitated from HEK293FT cells and the immunoprecipitate was analyzed for the presence of interacting proteins (Figure 3A). As previously reported, the E3 ubiquitin-protein ligase mouse double minute 2 (MDM2) was found in the AKT1 immunoprecipitate. This validates the immunoprecipitation conditions (32). Besides MDM2, the proteins UPF1, UPF3X, and the cap binding protein present during the pioneer round of translation CBP80, but not UPF2 or the cap binding protein present during the steady-state translation eIF4E, were detected in the immunoprecipitate. This suggests that AKT1 interacts with the NMD factors UPF1 and UPF3X. The presence of CBP80 but not eIF4E in the immunoprecipitate suggests that AKT1 interacts with NMD factors before or during the pioneer round of translation (33).

To confirm the putative interaction of AKT1 with NMD factors, immunoprecipitation of endogenous UPF1 was performed and the immunoprecipitate analyzed for the presence of interacting proteins (Figure 3B). While UPF2 and UPF3X, but not eIF4E, were detected in the UPF1 immunoprecipitate as previously reported (33,34), no AKT1 protein was detected under these conditions. To explain this discrepancy with respect to the results of Figure 3A, one might propose that the fraction of UPF1 protein interacting with AKT1 is low, so that the amount of AKT1 in the UPF1 immunoprecipitate is not detectable under these conditions. One way to increase the amount of AKT1 in the UPF1 immunoprecipitate is to increase the number of NMD events. For this, HEK293FT cells were transfected prior to UPF1 immunoprecipitation with the expression vector encoding Globin Ter mRNA (Figure 3C). Under these conditions, AKT1 was detected in the UPF1 immunoprecipitate, along with UPF2 and UPF3X but not eIF4E, as expected. Interestingly, when endogenous UPF3X immunoprecipitation was performed, UPF1, UPF2, and also AKT1 were immunoprecipitated with UPF3X, without the need to increase the number of NMD events. These results suggest that the interaction between AKT1 and UPF3X is more frequent than the interaction between AKT1 and UPF1 (Figure 3D). It should be noted that the interaction between AKT1 and UPF1 or UPF3X is dependent on the presence of RNA since RNase treatment prior to immunoprecipitation of endogenous AKT1, UPF1 or UPF3X partially prevented detection of interactions among these three proteins (Figure 3A, C and D). The efficiency of the RNase treatment was assessed by detecting the presence of RNA in the immunoprecipitate by RT-PCR (right panel of Figure 3C and D). Overall, these results suggest that these interactions take place on RNA and rarely or never, outside of mRNPs. As expected, interactions between UPF proteins were not affected by the absence of RNA, unlike interactions between CBP80 and UPF proteins (34,35).

**Figure 4.**
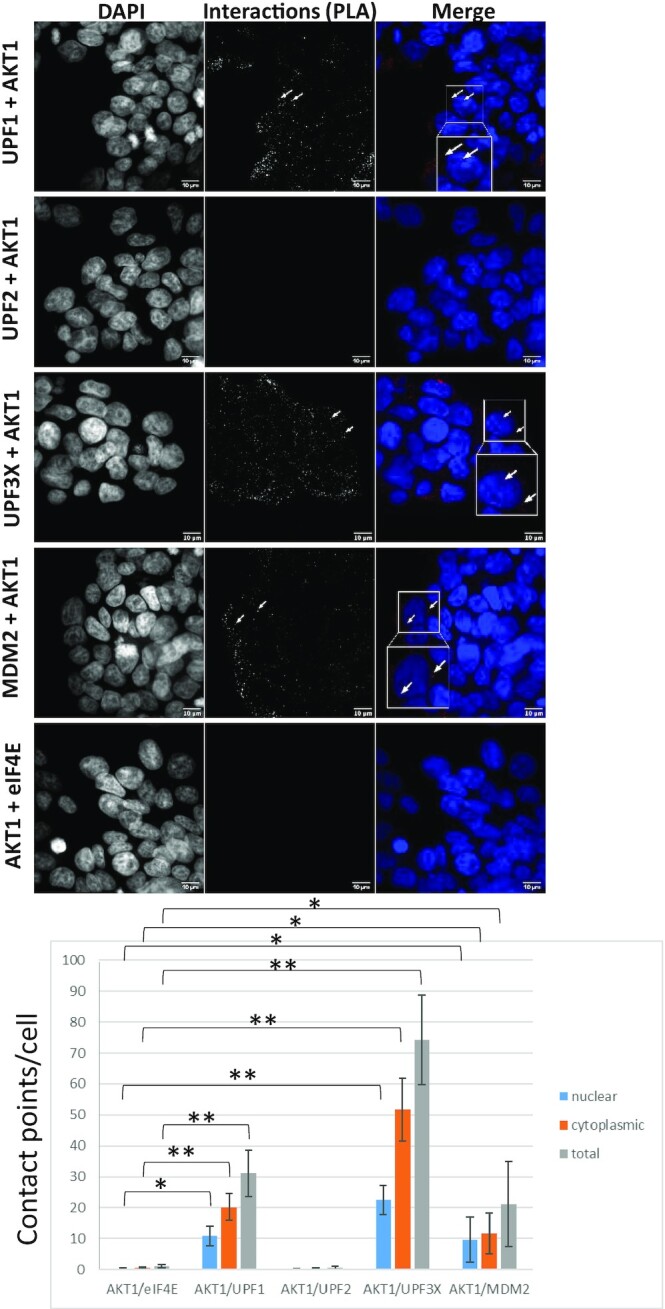
AKT1 interacts with UPF1 and UPF3X. A proximity ligation assay was performed to assess interactions between AKT1 and UPF1, UPF2, UPF3X or MDM2 (positive control). As a negative control, interactions between UPF1 and eIF4E were assessed. The white arrows indicate typical interaction points. The white squares correspond to a magnification of the background image. The bar plot at the bottom of the figure shows for each condition the average number of interaction points per cell as determined on more than 200 cells for each condition. Error bar = S.D., *P*-values were calculated with Student's *t*-test: *<0.05, **<0.01. All the results of this figure are representative of two experiments.

To further assess interactions between AKT1 and NMD factors under more physiological conditions, a proximity ligation assay (PLA) was performed (Figure 4) (36). The principle of this approach is that when the two studied proteins localize to within 40 nm of each other, a fluorescence signal is detected. This approach was validated by assessing the interaction between AKT1 and MDM2. As expected, several contact points per cell were detected. Contact points were also detected between AKT1 and UPF1 and between AKT1 and UPF3X, but not between AKT1 and UPF2 or AKT1 and eIF4E. These results are in total agreement with those presented in Figure 3. Interestingly, the contact points between AKT1 and UPF1 were observed mainly in the cytoplasm, like those between AKT1 and UPF3X. Between the latter two proteins, however, a significant proportion of the interactions was also found in the nucleus, suggesting that the interaction between AKT1 and UPF3X may happen earlier than the interaction between AKT1 and UPF1 (Figure 4). In agreement with the conclusions drawn from Figure 3, the average number of interactions observed between AKT1 and UPF3X was greater than the average number observed between AKT1 and UPF1.

**Figure 5.**
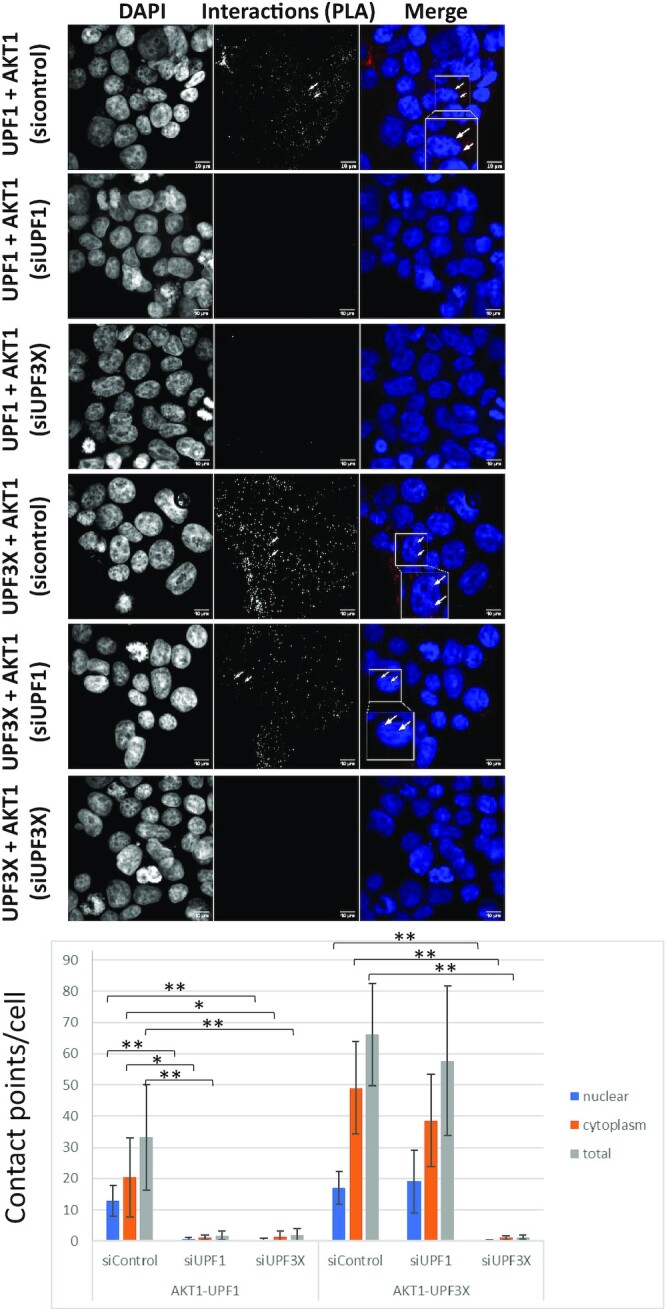
AKT1 interaction with UPF1 requires UPF3X. A proximity ligation assay was performed to assess the interactions between AKT1 and UPF1 or UPF3X after downregulation of UPF1 or UPF3X with siRNA. The white arrows indicate typical interaction points. The white squares correspond to a magnification of the background image. The bar plot at the bottom of the figure shows the average number of interaction points per cell as determined for >200 cells for each condition. Error bar = S.D., *P*-values were calculated with Student's *t*-test: *<0.05, **<0.01. All the results of this figure are representative of two experiments.

To understand the kinetics of interactions between AKT1 and UPF proteins, PLA was performed on cells where the level of UPF1 or UPF3X was reduced with an appropriate siRNA (Supplementary Figure S4). When UPF1 was downregulated, its interaction with AKT1 was lost, in contrast to the interaction between AKT1 and UPF3X. This indicates that UPF1 is not required for the interaction between AKT1 and UPF3X (Figure 5). When UPF3X was absent, on the other hand, the interaction between AKT1 and UPF3X was lost, naturally, but so was the interaction between AKT1 and UPF1. This suggests that UPF3X is required for the AKT1-UPF1 interaction. In addition, these results strengthen the idea that the interaction between AKT1 and UPF3X occurs before the interaction between AKT1 and UPF1 and that UPF3X is required for the interaction between AKT1 and UPF1. Consistently with the results of Figure 3 and with early recruitment of AKT1 by UPF3X, no interaction was detected by PLA between AKT1 and UPF2, and down-regulating UPF2 with siRNA did not affect the interaction between UPF3X and AKT1 (Supplementary Figure S5).

**Figure 6.**
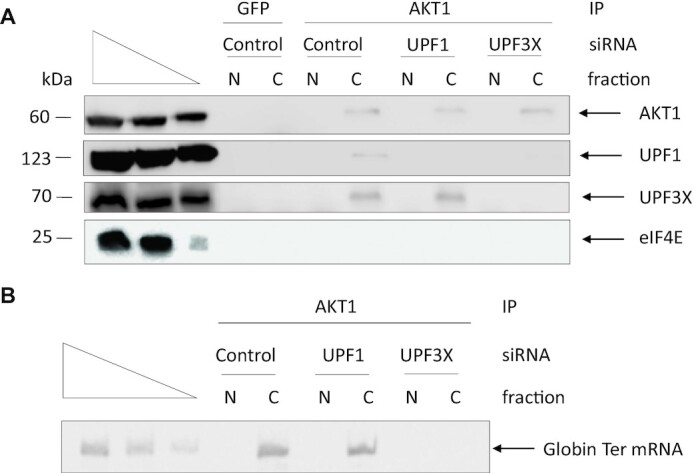
AKT1 is recruited to the mRNP by UPF3X. AKT1 immunoprecipitations were performed from nuclear and cytoplasmic fractions of HEK293FT WT cells transfected with a construct expressing Globin Ter mRNA. (**A**) Western blot analysis of the immunoprecipitates. The three leftmost lanes of each panel of the figure correspond to serial dilutions of HEK293FT whole cell extract. (**B**) RNAs were purified from the immunoprecipitates in order to measure the level of Globin Ter mRNA by RT-PCR. The three leftmost lanes correspond to serial dilutions of AKT1 immunoprecipitate from HEK293FT cells transfected with Control siRNA.

To confirm the results of PLA, AKT1 immunoprecipitations were performed on nuclear and cytoplasmic fractions from WT cells where UPF1 or UPF3X was downregulated with an siRNA and Globin Ter was introduced by transient transfection (Figure 6). Consistently with our PLA data (Figures 4 and 5) and although AKT1 is reported to be present in the nuclei of 293 cells (37), no AKT1 was detected in immunoprecipitates from nuclear fractions under any conditions tested (Figure 6A). Analysis of immunoprecipitates from the cytoplasmic fraction, however, revealed persistence of the interaction between AKT1 and UPF3X in the absence of UPF1. In contrast, UPF3X downregulation led to loss of the interaction between AKT1 and UPF1, consistently with our PLA results (Figure 5). RNA co-immunoprecipitation analysis revealed Globin Ter in AKT1 immunoprecipitates from cells transfected with control or UPF1 siRNA but not when UPF3X was downregulated. This indicates that the interaction between AKT1 and RNA requires UPF3X but not UPF1 (Figure 6B).

### AKT1 phosphorylates UPF1

**Figure 7.**
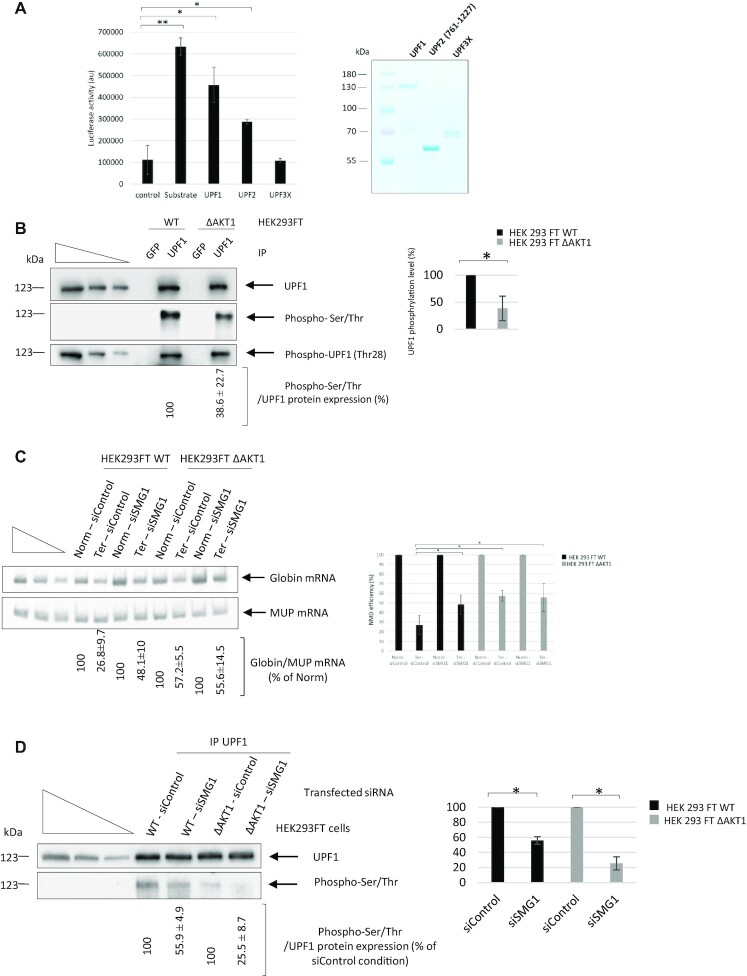
AKT1 phosphorylates NMD factors. (**A**) *In vitro* phosphorylation of UPF1 and UPF2 (761-1227) but not UPF3X by AKT1. Error bar = S.D., *P*-values were calculated with Student's *t*-test: *<0.05. The right panel is a Coomassie staining gel showing the UPF proteins used in the assay. (**B**) UPF1 was immunoprecipitated from HEK293FT WT and HEK293FT ΔAKT1 cells to assess the level of UPF1 phosphorylation. The three leftmost lanes of each panel of the figure correspond to serial dilutions of HEK293FT whole cell extract. The right panel shows quantification of UPF1 phosphorylation in both cell lines after immunoprecipitation of UPF1. (**C**) Absence of any additive effect of AKT1 and SMG1 on NMD efficiency. SMG1 was downregulated with siRNA in HEK293FT WT and HEK293FT ΔAKT1 cells expressing Globin Norm or Globin Ter in order to evaluate the NMD efficiency. The NMD efficiency was measured by RT-PCR. The left panel shows a representative gel analysis after quantitative PCR. MUP mRNA was used as a loading and transfection control. The three leftmost lanes correspond to serial dilutions of the Norm control plasmid sample. The bar plot on the right side of the gel shows the NMD efficiencies measured under the different tested conditions. (**D**) Measurement of UPF1 phosphorylation in HEK293FT WT and HEK293FT ΔAKT1 cells in the presence and absence of SMG1. The left panel shows a representative gel analysis and the bar plot on the right side of the gel is a graphic illustration of the quantification of two independent experiments. The three leftmost lanes of each panel of the figure correspond to serial dilutions of HEK293FT whole cell extract. Error bar = S.D., *P*-values were calculated with Student's t-test: *<0.05. The in vitro phosphorylation and immunoprecipitation results are representative of two and three experiments, respectively. The RT-PCR results showing the effects of SMG1 and AKT1 kinases are representative of four experiments.

Since UPF1 is a phosphoprotein and interacts with the kinase AKT1, the next step was to determine whether UPF1 might be phosphorylated by AKT1. This was tested in an *in vitro* phosphorylation assay using purified protein from bacteria (Figure 7A). Under these conditions, AKT1 was able to phosphorylate UPF1, UPF2 (761–1227), but not UPF3X. This indicates that the interaction between UPF3X and AKT1 detected by immunoprecipitation and in the proximity ligation assay does not lead to phosphorylation of UPF3X, in keeping with the fact that UPF3X has not been reported as a phosphoprotein.

To confirm the *in vitro* phosphorylation assay results, the phosphorylation level of UPF1 was assessed in the presence and absence of AKT1. UPF1 was immunoprecipitated from wild-type and ΔAKT1 cells and both the overall phosphorylation of UPF1 and the specific phosphorylation of threonine 28 were assessed. For this, either an anti-phosphorylated serine/threonine antibody or an anti-phosphorylated threonine 28 antibody was used (Figure 7B). HEK293FT ΔAKT1 cells showed an approximately two-fold lower overall UPF1 phosphorylation level than wild-type cells (Figure 7B, right panel), but the absence of AKT1 did not impair phosphorylation at threonine 28. This indicates that AKT1 does not phosphorylate this particular amino acid, usually phosphorylated by SMG1 (6). To determine the relative impacts of AKT1 and SMG1 downregulation on NMD efficiency, SMG1 was downregulated with siRNA (Supplementary Figure S4) in both cell lines (Figure 7C). When SMG1 was downregulated in HEK293FT WT cells, NMD was found to be significantly inhibited, consistently with previous reports (17). HEK293FT ΔAKT1 cells, interestingly, showed the same level of NMD efficiency whether SMG1 was downregulated or not. When the level of UPF1 phosphorylation was measured in UPF1 immunoprecipitates from extracts derived from WT or ΔAKT1 cells in the presence and absence of SMG1, wild-type cells showed an ∼50% decrease when SMG1 was absent (Figure 7D) and ΔAKT1 cells showed a very similar decrease (Figure 7B). Interestingly, when expression of both kinases, SMG1 and AKT1, was reduced, UPF1 phosphorylation dropped to an even lower level than when a single kinase was absent. This suggests that the two kinases have different phosphorylation sites on UPF1 (Figure 7D). Overall, the results of Figure 7 indicate that both kinases are required for NMD. To confirm the view that AKT1 is essential to NMD, the efficiency of NMD was measured in HEK293FT WT and HEK293FT ΔAKT1 cells in the presence of UPF1 and UPF3X and in the absence of one of these factors (Supplementary Figure S6). When the essential NMD factor UPF1 or UPF3X was absent, NMD was inhibited to the same extent as when AKT1 or SMG1 was absent. This indicates that AKT1 can also be considered a central factor in the NMD reaction.

### A hyperactivated AKT1 isoform enhances NMD efficiency

**Figure 8.**
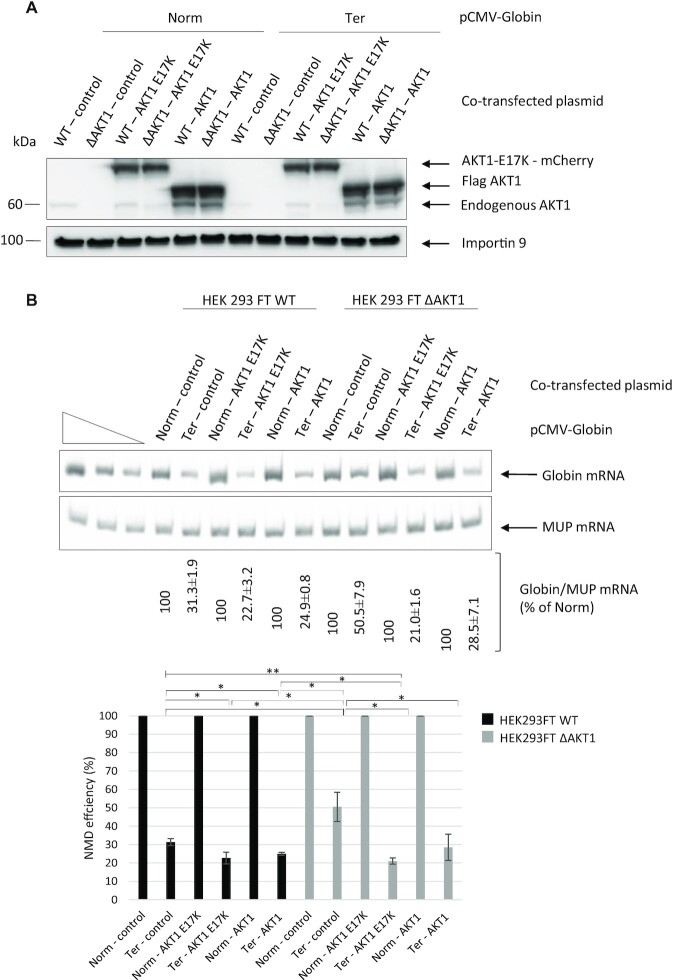
The NMD efficiency is related to the AKT1 activation status. (**A**) Western blot analysis showing levels of the endogenous, plasmid-encoded WT, and plasmid-encoded constitutively activated (E17K) AKT1 isoforms. Importin 9 was used as a loading control. (**B**) NMD efficiency measured by RT-PCR. The upper panel shows a representative gel analysis after quantitative PCR. MUP mRNA was used as a loading and transfection control. The three leftmost lanes correspond to serial dilutions of the Norm control plasmid sample. The bar plot at the bottom of the figure shows the NMD efficiencies measured under the different test conditions. Error bar = S.D., *P*-values were calculated with Student's *t*-test: *<0.05, **<0.01. All the results of this figure are representative of three experiments.

Some somatic mutations in the *AKT1* gene are reported to be associated with various cancers, including breast, colorectal and ovarian cancers. Among these mutations, a glutamic-acid-to-lysine substitution at position 17 promotes hyperactivation of AKT1 due to increased AKT1 phosphorylation, resulting in concentration of this isoform at the cell membrane (38,39). When the E17K AKT1 isoform was expressed along with the Globin Norm or Globin Ter construct in transfected WT or HEK293FT ΔAKT1 cells, both cell lines showed a significant increase in NMD efficiency (Figure 8). This effect was even greater than that obtained with overexpression of the wild-type isoform, although the levels of the two proteins were similar. Overall, the results in Figure 8 show that the NMD efficiency may be related to the level of AKT1 activation.

## DISCUSSION

**Figure 9.**
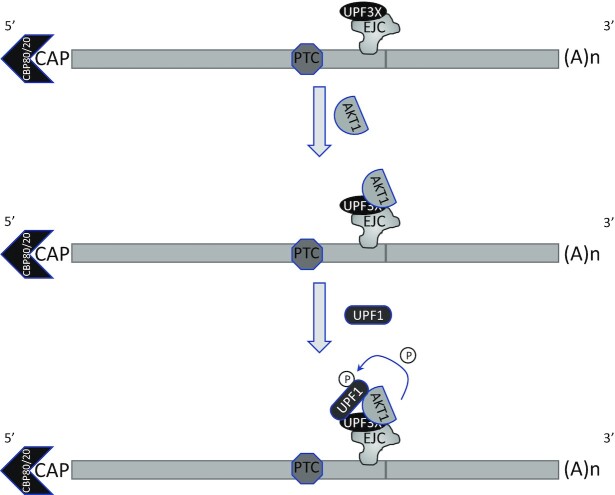
Model of UPF1 phosphorylation by AKT1. UPF3X is loaded on mRNP by the exon junction complex (EJC). UPF3X would recruit AKT1 before recruiting UPF1 and phosphorylating it. These events occur before or during the pioneer round of translation, since the cap structure is bound by CBP80 and not eIF4E.

AKT is a kinase involved in the PI3K/AKT/mTOR signaling pathway. Targets of this kinase participate in many cell processes, including apoptosis, cell proliferation and cell survival (40). An indirect involvement of AKT in NMD has been suggested previously: via activation of the mammalian target of rapamycin complex 1 (mTORC1), AKT might cause phosphorylation of eIF4E-BP resulting in activation of translation and NMD (41). Yet, it seems very likely that instead, AKT1 plays a direct role in NMD via UPF1 phosphorylation. The screening method used at the start of this work does not allow identification of NMD inhibitors whose mode of action involves inhibition of translation, since the readout is a measure of the activity of newly synthesized luciferase (Figure 1). Interestingly, all three molecules identified, in our screen of a library of kinase inhibitors, as having the greatest NMD-inhibiting capacity target the same kinase: AKT1. This led us to validate the involvement of AKT1 in NMD (Figure 2) and to demonstrate interactions between AKT1 and both UPF1 and UPF3X (Figures 3 and 4). The initial evidence that AKT1 is recruited to the mRNP by UPF3X before recruitment of UPF1 is supported by our PLA and immunoprecipitation results obtained in the absence of UPF1 or UPF3X (Figures 5 and 6). In addition, the presence of CBP80, but not eIF4E, in the AKT1 immunoprecipitate indicates that AKT1 is recruited to mRNP before or during the pioneer round of translation (33). Although AKT1 interacts with UPF1 and UPF3X, only UPF1 is phosphorylated by AKT1 (Figure 7). All these results can be summarized by the model described in Figure 9. The recruitment of AKT1 by UPF3X suggests that the AKT1 kinase might not be involved in all NMD reactions, particularly those which are independent of the UPF3X factor (42).

AKT1, shown here to phosphorylate UPF1 directly, is the second protein kinase, after SMG1 (16,17), to be identified as involved in NMD. Two other phosphorylation events are reported to influence NMD efficiency by targeting proteins indirectly involved in NMD. The first is phosphorylation of the telomere-maintenance 2 protein (TL2) by casein kinase 2 (CK2) and the second is phosphorylation of the exon junction complex component eIF4A3 by cyclin dependent kinase (43,44). Results presented in this study demonstrate that AKT1 is as essential to NMD as SMG1 (Figure 7C). Interestingly, these two kinases do not fully recognize the same phosphorylation sites on UPF1 but could rather recognize only some or on the contrary totally different sites (Figure 7). This suggests a complex regulation of NMD by these two kinases, to be elucidated in the future. We have already shown that NMD is more efficient in cells where AKT1 is constitutively activated. This activation of NMD, allowing elimination of PTC-carrying mutant mRNAs, might have an important selective advantage in tumorigenesis. In fact, the number of NMD reactions increases in cancer cells because of the accumulation of neo-mutations at each cell division (45,46).

The involvement of AKT1 in NMD constitutes a link between NMD and the PI3K/AKT/mTOR signaling pathway and potentially between NMD and the many processes in which AKT1 intervenes. In particular, this signaling pathway is very often activated during tumorigenesis, AKT being hyperactivated in more than 50% of tumors (40). On the basis of the results presented here, one might tentatively suggest that one consequence of AKT1 hyperactivation during tumorigenesis is activation of NMD. This view may be somewhat controversial, as several reports indicate inhibition of NMD in cancer cells, due to inhibition of NMD regulators such as MARVELD1 and UPF1 through promoter hypermethylation (47–49). This, once again, highlights the complexity of tumorigenesis. To fully understand this process, we need to understand the molecular mechanisms involved and the mutations promoting it. It seems fairly clear that according to the mutation(s) inducing or involved in the progression of tumorigenesis, NMD will be activated or inhibited and can play a protective role or even amplify tumorigenesis (4,50). Apart from this, the role of NMD may also differ according to the type of cancer. For example, it has been demonstrated that NMD is activated in cancers with microsatellite instability, particularly because of increased expression of some of the factors involved in NMD (51).

Whatever the case may be, the involvement of AKT1 in NMD might open interesting therapeutic prospects. Several AKT inhibitors have been developed for anticancer therapy, with promising results. Examples include AZD5363, Afuresertib and Ipatasertib (52–54). Inhibition of NMD might be one of the action mechanisms of such inhibitors, as reported for Rigosertib (55). Furthermore, NMD inhibition has been proposed as a therapeutic approach for cancers (56), and this inhibition might be achieved with AKT1 inhibitors.

## DATA AVAILABILITY

Data and materials are fully available on request.

## Supplementary Material

gkab882_Supplemental_File
