# Supplementary material for: A role for AKT1 in nonsense-mediated mRNA decay
Source: Nucleic Acids Res. 2021 Oct 11;49(19):11022–37. doi: 10.1093/nar/gkab882 (PMC8565340; doi:10.1093/nar/gkab882)
Supplement: gkab882_Supplemental_File [file gkab882_supplemental_file.pdf]

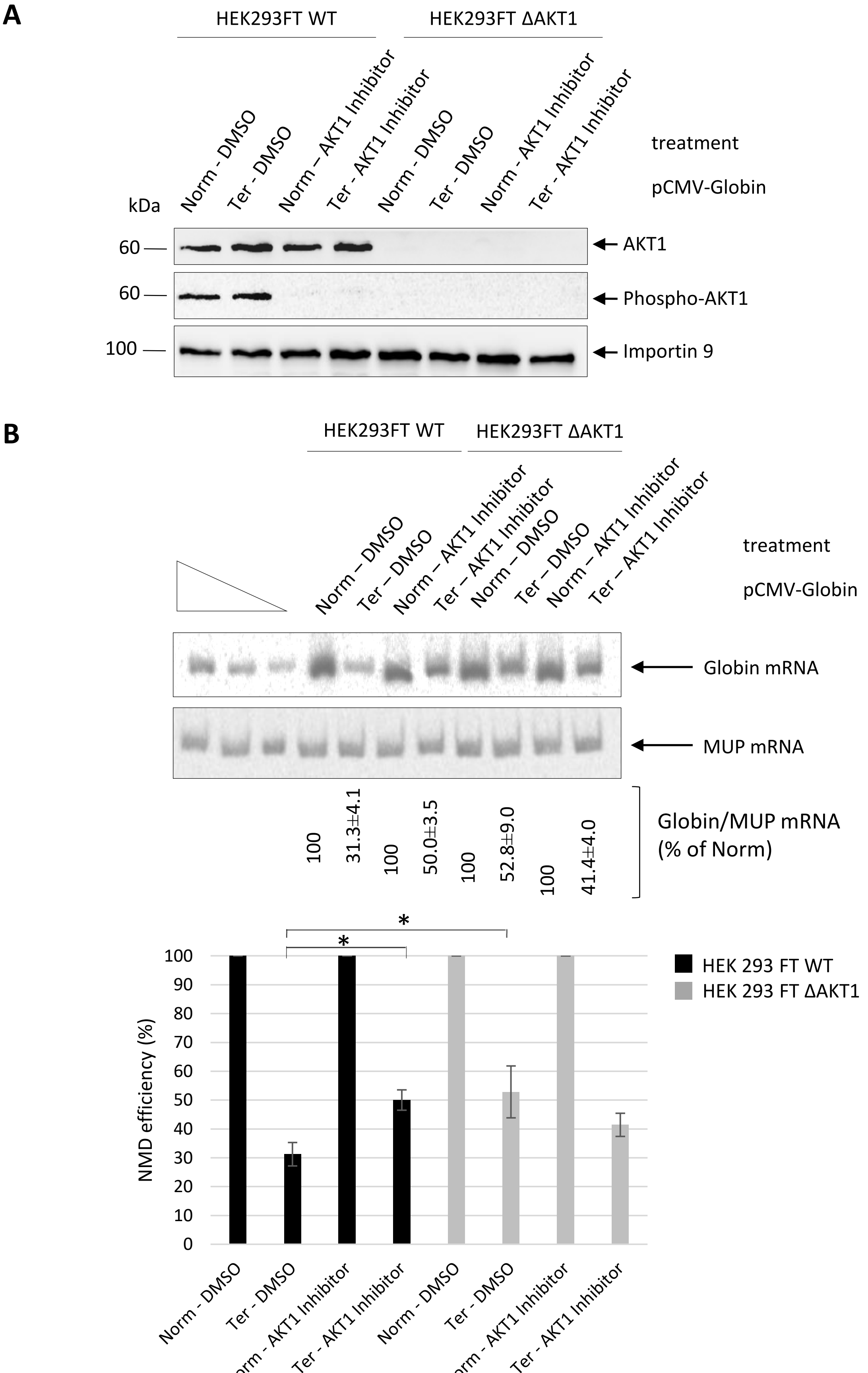

Supplemental Figure 1: Chemical inhibition of AKT1 promotes NMD inhibition. (A) Western blot analysis of proteins from HEK293FT WT and HEK293FT DAKT1 cells treated with DMSO or with the AKT1 inhibitor. AKT1 and phospho-AKT1 (P-AKT1) were detected. Importin 9 was used as a loading control. (B) RT-PCR analysis of HEK293FT WT and HEK293FT DAKT1 cells transfected with the Globin Norm or Globin Ter expression vector and treated with DMSO or the AKT1 inhibitor. The three leftmost lanes correspond to serial dilutions of the Norm control plasmid sample. The bar plot at the bottom of the figure shows the NMD efficiencies measured under the different test conditions. Error bar= S.D., p-values were calculated with Student's t-test: *<0.05. All the results of this figure are representative of three experiments.

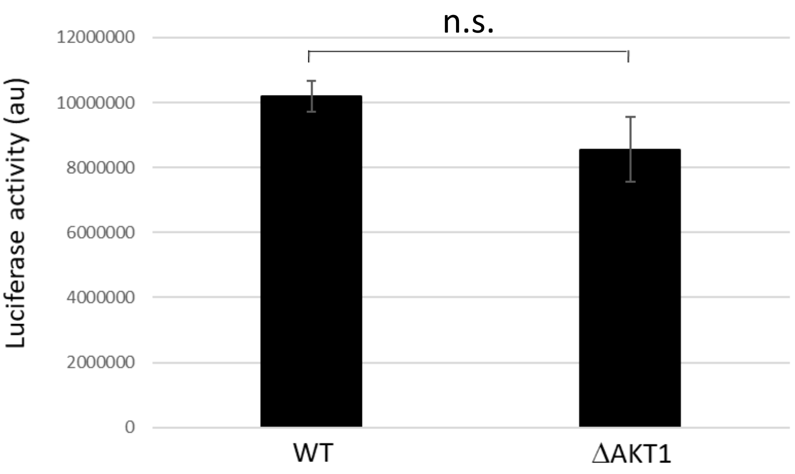

Supplemental Figure 2: Measurement of luciferase activity in HEK293FT WT and HEK293FT DAKT1 cells. These cells were transfected with an expression vector encoding firefly luciferase. Twenty-four hours after transfection, the cells were counted and 70,000 cells were plated in wells of a 96-well plate. Twenty-four hours after, the luciferase activity was measured with a luminometer after adding steadylite luciferase substrate (Perkin Elmer). n.s.: non-significant difference. Results of this figure come from independent triplicates.

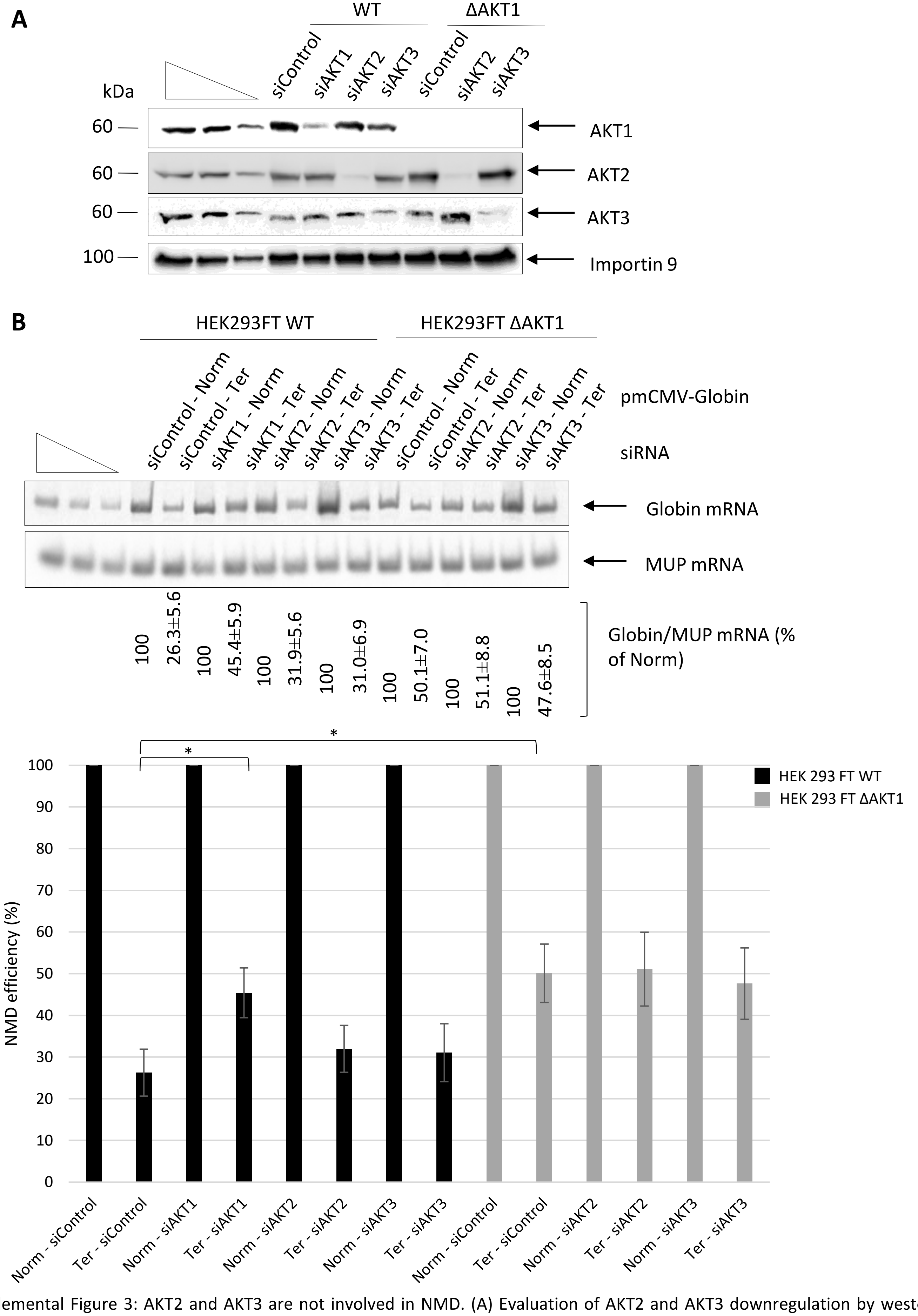

Supplemental Figure 3: AKT2 and AKT3 are not involved in NMD. (A) Evaluation of AKT2 and AKT3 downregulation by western blotting. HEK293FT WT and HEK293FT DAKT1 cells were transfected with an siRNA against AKT2 or AKT3 mRNA. The three leftmost lanes correspond to serial dilutions of HEK293FT whole cell extract. Importin 9 was used as a loading control. (B) NMD efficiency was measured by RT-PCR in HEK293FT WT and HEK293FT DAKT1 cells transfected with a nonspecific (control), AKT2-targeting, or AKT3-targeting siRNA and with pmCMV-Globin Norm or pmCMV-Globin Ter. MUP mRNA was used as a loading and transfection control. The three leftmost lanes correspond to serial dilutions of the Globin Norm sample. Error bar= S.D., p-values were calculated with Student's t-test: *<0.05. All the results of this figure are representative of four experiments.

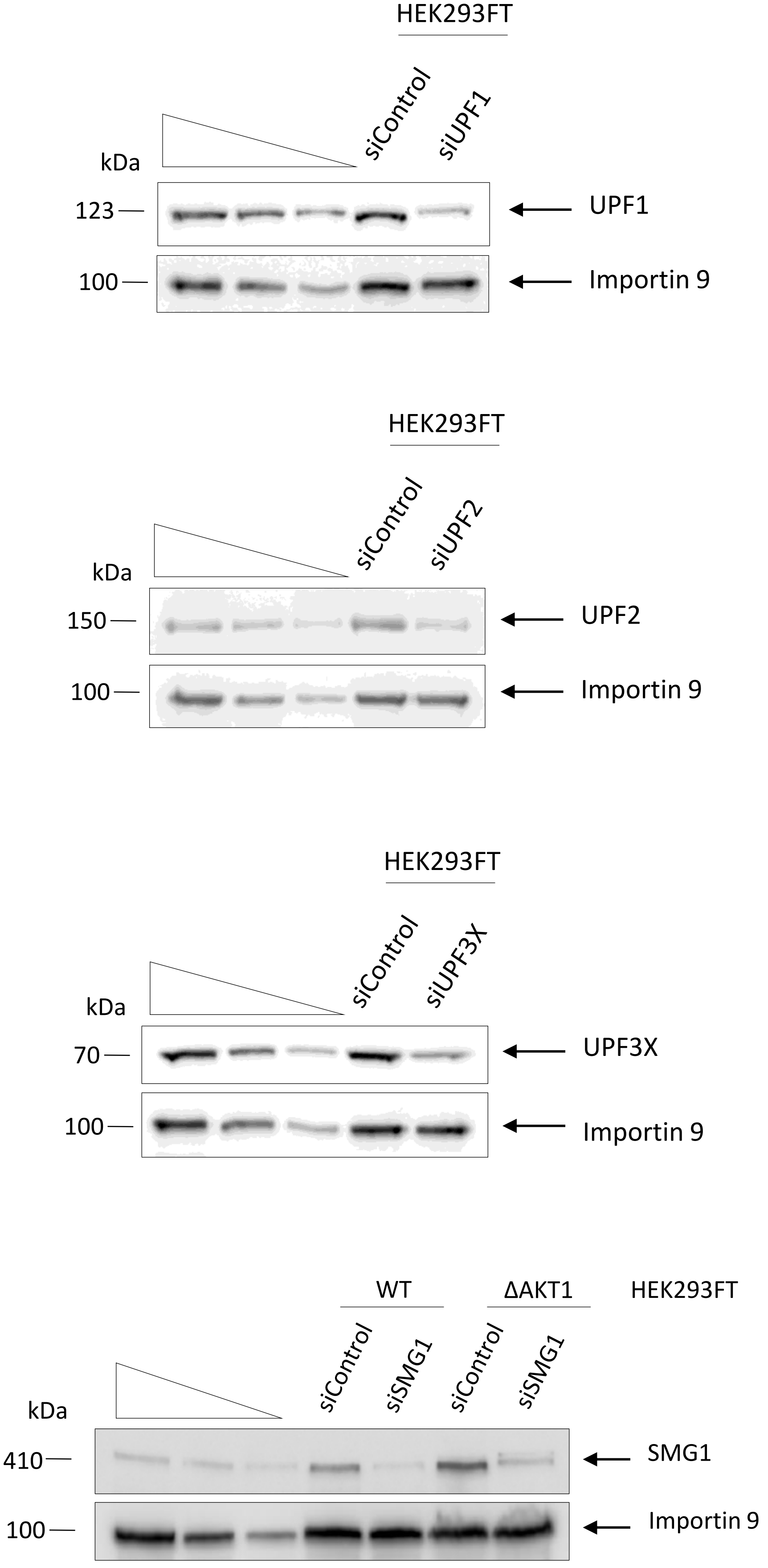

Supplemental Figure 4: Western blot analysis showing downregulation of UPF1 (upper panel), UPF2 (second panel from the top), UPF3X (third panel from the top), and SMG1 (lower panel) by targeting siRNAs in HEK293FT WT and HEK293FT DAKT1 cells. The three leftmost lanes correspond to serial dilutions of HEK293FT WT whole cell extract. Importin 9 was used as a loading control.

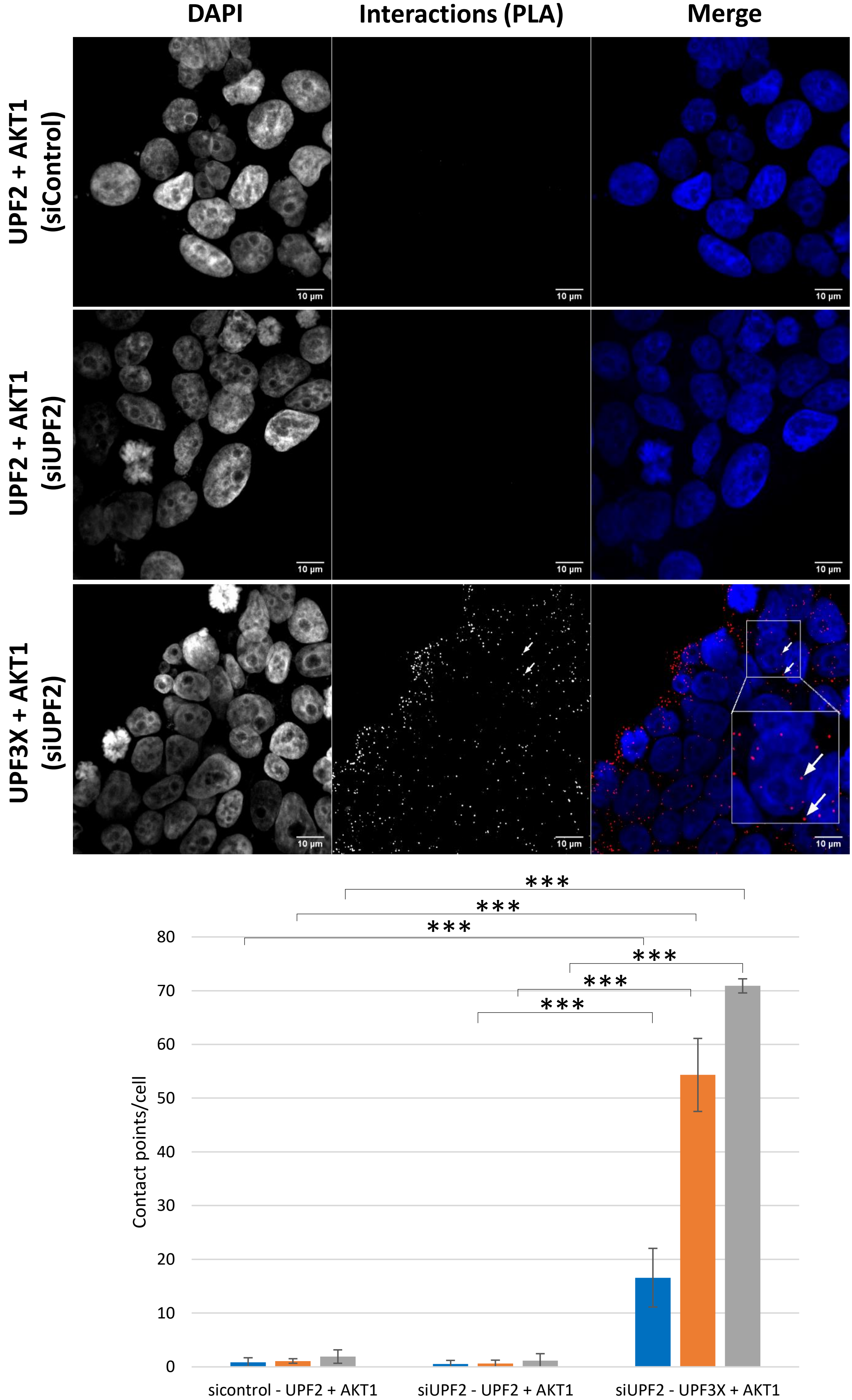

Supplemental Figure 5: UPF2 is not required for the interaction between UPF3X and AKT1. A proximity ligation assay was performed to assess interactions between AKT1 and UPF2 or UPF3X in the presence and absence of UPF2. White arrows indicate typical interaction points. White squares correspond to a magnification of the background image. The bar plot at the bottom of the figure shows for each condition the average number of interaction points per cell as determined on more than 200 cells for each condition. Error bar=S.D., p-values were calculated with Student's t-test: *<0.05, **<0.01, ***<0.001. All the results of this figure are representative of two experiments.

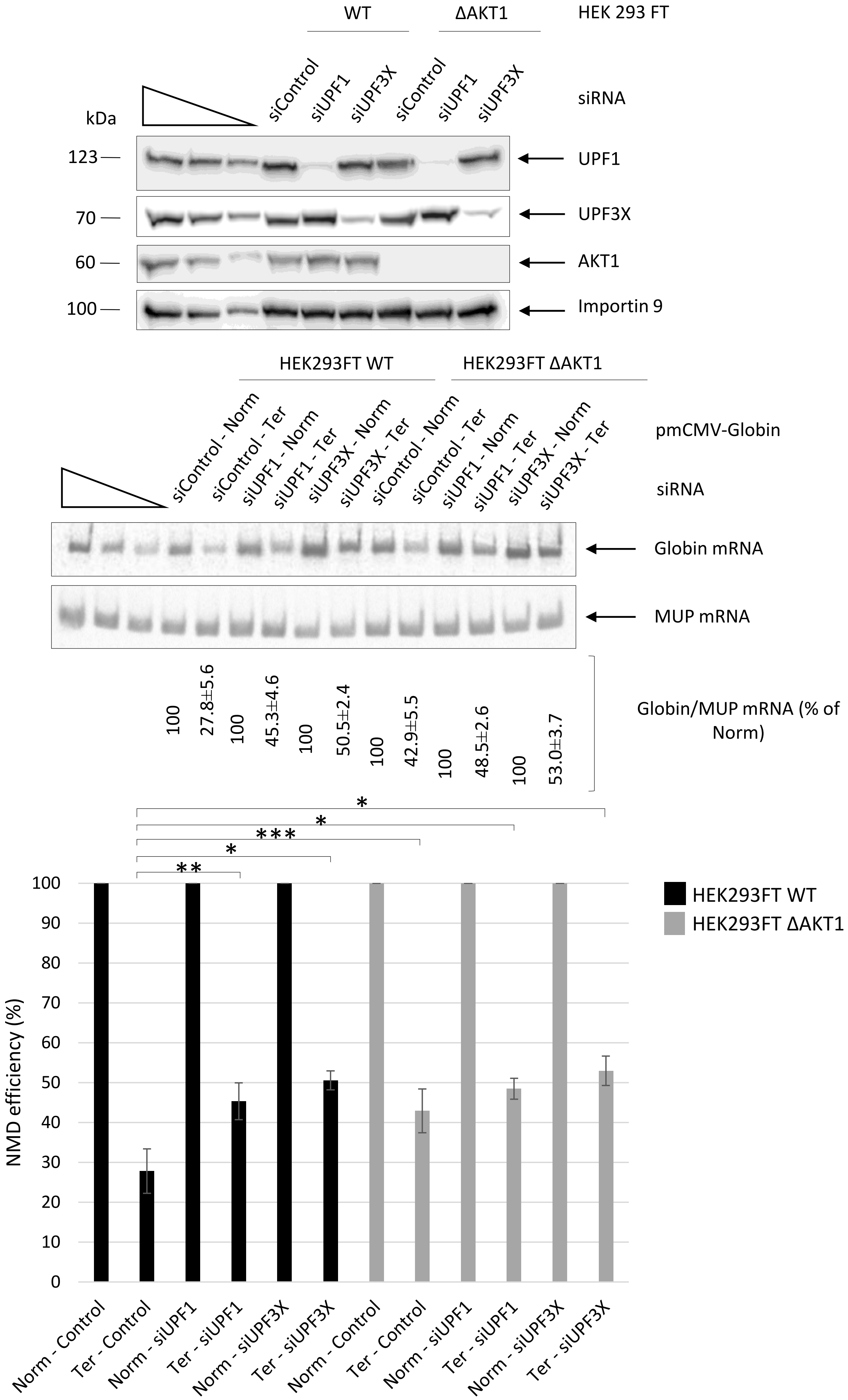

WT
ΔAKT1
HEK 293 FT
siControl
siUPF1
siUPF3X
siControl
siUPF1
siUPF3X
siRNA
kDa
123
UPF1
70
UPF3X
60
AKT1
100
Importin 9
HEK293FT WT
HEK293FT ΔAKT1
siControl - Norm
siControl - Ter
siUPF1 - Norm
siUPF1 - Ter
siUPF3X - Norm
siUPF3X - Ter
siControl - Norm
siControl - Ter
siUPF1 - Norm
siUPF1 - Ter
siUPF3X - Norm
siUPF3X - Ter
pmCMV-Globin
siRNA
Globin mRNA
MUP mRNA
100
27.8±5.6
100
45.3±4.6
100
50.5±2.4
100
42.9±5.5
100
48.5±2.6
100
53.0±3.7
Globin/MUP mRNA (% of Norm)
*
*
***
*
**
NMD efficiency (%)
0
10
20
30
40
50
60
70
80
90
100
HEK293FT WT
HEK293FT ΔAKT1
Norm - Control
Ter - Control
Norm - siUPF1
Ter - siUPF1
Norm - siUPF3X
Ter - siUPF3X
Norm - Control
Ter - Control
Norm - siUPF1
Ter - siUPF1
Norm - siUPF3X
Ter - siUPF3X

Supplemental Figure 6: Measure of NMD inhibition in HEK293FT WT and HEK293FT DAKT1 cells in the presence and absence of UPF1 or UPF3X. (Upper panel). Western blot showing downregulation of UPF1 or UPF3X after siRNA treatment of the cells and the level of AKT1 in both cell lines. The molecular weight of each protein is indicated on the left side of the gels. The three leftmost lanes correspond to serial dilutions of HEK293FT WT whole cell extract. Importin 9 was used as a loading control. (Middle and lower panels) RT-PCR analysis performed to measure the NMD efficiency in the presence of UPF1 and UPF3X and in the absence of one of these in both cell lines. The three leftmost lanes correspond to serial dilutions of the Globin Norm sample. Error bar= S.D., p-values were calculated with Student's t-test: *<0.05, **<0.01, ***<0.001. All the results of this figure are representative of two experiments.
